# Supplementary material for: A Randomised Trial to Optimise Gestational Weight Gain and Improve Maternal and Infant Health Outcomes through Antenatal Dietary, Lifestyle and Exercise Advice: The OPTIMISE Randomised Trial
Source: Nutrients. 2019 Dec 2;11(12):2911. doi: 10.3390/nu11122911 (PMC6949931; doi:10.3390/nu11122911)
Supplement: Supplementary file 1 [file nutrients-11-02911-s001.pdf]

nutrients

MDPI

Table S1. Maternal Quality of Life.

Outcome	Lifestyle Advice (*n* = 316) **	Standard Care (*n* = 313) **	Unadjusted Estimate (95% CI)	Unadj. *p* Value	Adjusted Estimate (95% CI) [f]	Adj. *p* Value
SF-12 Physical Score [b, e]				0.185 *		0.185 *
Trial Entry	50.70 (9.82)	51.80 (9.21)	−1.10 (−2.60, 0.39)	0.149	−1.13 (-2.64, 0.38)	0.143
28 Weeks	46.38 (9.99)	46.03 (10.21)	0.35 (-1.24, 1.94)	0.667	0.32 (-1.27, 1.91)	0.689
36 weeks	41.20 (12.66)	41.70 (11.12)	-0.49 (-2.37, 1.39)	0.608	-0.52 (-2.39, 1.36)	0.589
SF-12 Mental Score [b, e]				0.116 *		0.116*
Trial Entry	41.31 (10.74)	41.62 (10.39)	-0.31 (-1.97, 1.35)	0.716	-0.33 (-2.00, 1.34)	0.699
28 Weeks	46.33 (10.05)	45.08 (9.96)	1.25 (-0.31, 2.82)	0.117	1.23 (-0.33, 2.80)	0.122
36 weeks	46.26 (9.91)	46.15 (9.49)	0.10 (-1.40, 1.60)	0.892	0.08 (-1.41, 1.58)	0.913
EPDS Total Score[b,e]				0.055*		0.055*
Trial Entry	5.03 (4.40)	5.11 (4.41)	-0.08 (-0.77, 0.60)	0.814	-0.05 (-0.74, 0.64)	0.884
28 Weeks	5.59 (4.20)	6.09 (4.84)	-0.51 (-1.21, 0.20)	0.159	-0.48 (-1.18, 0.23)	0.187
36 weeks	5.35 (4.19)	5.17 (4.45)	0.18 (-0.50, 0.86)	0.599	0.21 (-0.47, 0.89)	0.539
EPDS > 12 [a, e]				0.259*		0.259*
Trial Entry	24 (7.72)	20 (6.45)	1.20 (0.67, 2.12)	0.541	1.20 (0.68, 2.14)	0.529
28 Weeks	19 (5.96)	21 (6.84)	0.87 (0.46, 1.65)	0.673	0.88 (0.46, 1.67)	0.690
36 Weeks	22 (6.84)	13 (4.06)	1.69 (0.80, 3.58)	0.173	1.70 (0.80, 3.63)	0.170
STAI Score [b, e]				0.814*		0.814*
Trial Entry	10.35 (3.46)	10.70 (3.70)	-0.35 (-0.92, 0.21)	0.220	-0.34 (-0.90, 0.23)	0.244
28 Weeks	9.87 (3.46)	10.39 (3.61)	-0.52 (-1.07, 0.04)	0.068	-0.50 (-1.06, 0.05)	0.077
36 weeks	9.90 (3.28)	10.33 (3.67)	-0.43 (-0.97, 0.12)	0.123	-0.41 (-0.95, 0.13)	0.135
STAI > 15 [a, e]				0.563*		0.564*
Trial Entry	23 (7.42)	27 (8.49)	0.87 (0.49, 1.54)	0.639	0.88 (0.49, 1.55)	0.649
28 Weeks	21 (6.68)	26 (8.17)	0.81 (0.45, 1.48)	0.502	0.82 (0.45, 1.49)	0.510
36 Weeks	15 (4.71)	26 (8.45)	0.55 (0.28, 1.09)	0.088	0.56 (0.28, 1.09)	0.089

[a] number and percentage, estimates are Relative Risks and 95% Confidence Interval. [b] mean and standard deviation, estimates are differences in means and 95% Confidence Interval. [e] Repeated Measures Outcomes: models included a time by intervention interaction term, and separate estimates of treatment effect were derived at each time point regardless of the significance of this interaction term. [f] Adjusted analyses: all outcomes were adjusted for the stratification variable parity

(0 vs 1+), maternal age (continuous), maternal pre-pregnancy BMI (continuous), and SEIFA IRSD Quintile. * Denotes p value testing for interaction between treatment and time, i.e. whether effect of intervention differed between time points. **=Includes all women randomized who did not withdraw consent to use their data, and who did not suffer miscarriage or termination of pregnancy prior to 20 weeks gestation, or stillbirth.

Table S2. Infant anthropometry.

Outcome	Lifestyle Advice (n=316)**	Standard Care (n=313)**	Unadjusted Estimate (95% CI)	Unadj. p value	Adjusted Estimate (95% CI)[c]	Adj. p value
Birth Length (cm)[b]	49.29 (3.19)	49.60 (2.48)	-0.31 (-0.76, 0.13)	0.170	-0.32 (-0.77, 0.13)	0.166
Birth Length z-score[b]	-0.40 (0.78)	-0.42 (0.78)	0.02 (-0.10, 0.14)	0.784	0.02 (-0.11, 0.14)	0.785
Birth Head C (cm)[b]	34.29 (1.94)	34.56 (1.63)	-0.28 (-0.56, 0.00)	0.053	-0.28 (-0.56, 0.00)	0.053
Birth Head C z-score[b]	-0.09 (1.01)	-0.04 (1.06)	-0.05 (-0.21, 0.11)	0.547	-0.05 (-0.21, 0.11)	0.568
Abdomen C (cm)[b]	32.61 (3.16)	32.88 (2.69)	-0.27 (-0.73, 0.18)	0.241	-0.26 (-0.71, 0.19)	0.251
Chest C (cm)[b]	33.83 (2.73)	34.18 (2.47)	-0.35 (-0.75, 0.05)	0.086	-0.36 (-0.76, 0.03)	0.073
Arm C (cm)[b]	10.72 (1.16)	10.86 (1.23)	-0.15 (-0.33, 0.04)	0.124	-0.14 (-0.33, 0.04)	0.131
Biceps SFTM (mm)[b]	4.51 (1.58)	4.44 (1.45)	0.07 (-0.17, 0.31)	0.583	0.05 (-0.19, 0.29)	0.667
Triceps SFTM (mm)[b]	5.53 (2.02)	5.70 (2.00)	-0.17 (-0.48, 0.15)	0.296	-0.18 (-0.49, 0.13)	0.263
Subscapular SFTM (mm)[b]	4.98 (1.51)	5.14 (1.50)	-0.16 (-0.40, 0.07)	0.174	-0.17 (-0.41, 0.06)	0.151
Suprailiac SFTM (mm)[b]	4.47 (1.50)	4.52 (1.62)	-0.05 (-0.30, 0.20)	0.688	-0.06 (-0.31, 0.18)	0.624
Abdomen SFTM (mm)[b]	3.91 (1.29)	3.99 (1.24)	-0.09 (-0.28, 0.11)	0.375	-0.09 (-0.28, 0.11)	0.389
Thigh SFTM (mm)[b]	6.88 (2.01)	7.16 (2.36)	-0.28 (-0.62, 0.06)	0.109	-0.29 (-0.63, 0.04)	0.088

[b] mean and standard deviation, estimates are differences in means and 95% Confidence Interval. [f] Adjusted analyses: all outcomes were adjusted for the stratification variable parity (0 vs 1+), maternal age (continuous), maternal pre-pregnancy BMI (continuous), and SEIFA IRSD Quintile. **=Includes all infants of women randomized who did not withdraw consent to use their data, and who did not suffer miscarriage or termination of pregnancy prior to 20 weeks gestation, or stillbirth.

Table S3. Pre-specified analysis of effect modification by maternal pre-pregnancy BMI.

Outcome	Lifestyle Advice (n=316)**	Standard Care (n=313)**	Unadjusted Estimate (95% CI)	Unadj. p value	Adjusted Estimate (95% CI)[c]	Adj. p value
Birthweight > 4kg[a]				0.230*		0.263*
- BMI At Mean	12 (7.45)	10 (6.34)	0.94 (0.55, 1.62)	0.835	0.94 (0.55, 1.62)	0.836
- BMI +1 Unit	12 (7.74)	16 (10.32)	0.77 (0.43, 1.41)	0.404	0.79 (0.44, 1.43)	0.434
Birthweight (g)[b]				0.030*		0.024*
- BMI At Mean	3288.58 (555.80)	3296.62 (472.34)	-79.51 (-165.02, 6.00)	0.068	-78.11 (-163.44, 7.22)	0.073
- BMI +1 Unit	3295.48 (617.97)	3446.66 (539.10)	-135.42 (-234.72, -36.12)	0.008	-135.83 (-234.75, -36.92)	0.007
Birthweight z-score[b]				0.045*		0.036*
- BMI At Mean	-0.02 (0.87)	-0.09 (0.84)	-0.05 (-0.18, 0.09)	0.492	-0.04 (-0.18, 0.09)	0.535
- BMI +1 Unit	0.00 (0.89)	0.16 (0.92)	-0.13 (-0.29, 0.03)	0.108	-0.13 (-0.29, 0.03)	0.109
Birth Length (cm)[b]				0.184*		0.183*
- BMI At Mean	49.36 (2.97)	49.42 (2.29)	-0.31 (-0.76, 0.13)	0.168	-0.32 (-0.77, 0.13)	0.167
- BMI +1 Unit	49.21 (3.42)	49.78 (2.65)	-0.49 (-1.01, 0.03)	0.063	-0.50 (-1.02, 0.02)	0.062
Birth Length z-score[b]				0.361*		0.370*
- BMI At Mean	-0.38 (0.75)	-0.45 (0.74)	0.02 (-0.10, 0.14)	0.785	0.02 (-0.10, 0.14)	0.783
- BMI +1 Unit	-0.42 (0.80)	-0.38 (0.82)	-0.02 (-0.16, 0.12)	0.818	-0.02 (-0.16, 0.13)	0.828
Birth HC (cm)[b]				0.083*		0.081*
- BMI At Mean	34.28 (1.82)	34.44 (1.54)	-0.28 (-0.56, 0.00)	0.051	-0.28 (-0.56, 0.00)	0.053
- BMI +1 Unit	34.30 (2.07)	34.69 (1.70)	-0.42 (-0.75, -0.10)	0.010	-0.42 (-0.75, -0.10)	0.011
Birth HC z-score[b]				0.123*		0.122*
- BMI At Mean	-0.10 (0.98)	-0.11 (1.09)	-0.05 (-0.21, 0.11)	0.542	-0.05 (-0.21, 0.11)	0.571
- BMI +1 Unit	-0.08 (1.05)	0.03 (1.03)	-0.13 (-0.31, 0.06)	0.191	-0.12 (-0.31, 0.07)	0.203
Abdomen C (cm)[b]				0.053*		0.040*
- BMI At Mean	32.68 (2.99)	32.60 (2.58)	-0.27 (-0.73, 0.18)	0.236	-0.26 (-0.71, 0.19)	0.252
- BMI +1 Unit	32.54 (3.19)	33.17 (2.79)	-0.54 (-1.06, -0.01)	0.047	-0.54 (-1.06, -0.01)	0.044
Chest C (cm)[b]				0.013*		0.012*
- BMI At Mean	33.82 (2.49)	33.78 (2.37)	-0.35 (-0.75, 0.04)	0.081	-0.36 (-0.76, 0.03)	0.073
- BMI +1 Unit	33.83 (2.85)	34.59 (2.44)	-0.65 (-1.11, -0.19)	0.006	-0.66 (-1.12, -0.20)	0.005
Arm C (cm)[b]				0.723*		0.679*
- BMI At Mean	10.55 (1.03)	10.68 (1.19)	-0.15 (-0.33, 0.04)	0.115	-0.14 (-0.33, 0.04)	0.132

- BMI +1 Unit	10.89 (1.22)	11.05 (1.18)	-0.17 (-0.38, 0.05)	0.122	-0.16 (-0.38, 0.05)	0.130
Biceps SFTM (mm)[b]				0.868*		0.821*
- BMI At Mean	4.45 (1.44)	4.43 (1.57)	0.07 (-0.17, 0.31)	0.586	0.05 (-0.19, 0.29)	0.667
- BMI +1 Unit	4.57 (1.63)	4.45 (1.30)	0.08 (-0.20, 0.36)	0.579	0.07 (-0.21, 0.35)	0.627
Triceps SFTM (mm)[b]				0.991*		0.999*
- BMI At Mean	5.40 (1.79)	5.58 (2.05)	-0.17 (-0.48, 0.14)	0.289	-0.18 (-0.49, 0.13)	0.263
- BMI +1 Unit	5.66 (2.23)	5.82 (1.88)	-0.17 (-0.54, 0.20)	0.361	-0.18 (-0.55, 0.19)	0.336
Subscapular SFTM (mm)[b]				0.263*		0.266*
- BMI At Mean	4.97 (1.40)	5.02 (1.47)	-0.17 (-0.40, 0.07)	0.170	-0.17 (-0.41, 0.06)	0.152
- BMI +1 Unit	4.99 (1.49)	5.27 (1.49)	-0.24 (-0.52, 0.03)	0.083	-0.25 (-0.52, 0.03)	0.075
Suprailiac SFTM (mm)[b]				0.608*		0.578*
- BMI At Mean	4.45 (1.38)	4.53 (1.70)	-0.05 (-0.30, 0.20)	0.686	-0.06 (-0.31, 0.18)	0.623
- BMI +1 Unit	4.48 (1.56)	4.51 (1.45)	-0.01 (-0.29, 0.26)	0.920	-0.02 (-0.30, 0.25)	0.876
Abdomen SFTM (mm)[b]				0.627*		0.595*
- BMI At Mean	3.95 (1.25)	3.97 (1.30)	-0.09 (-0.28, 0.11)	0.375	-0.09 (-0.28, 0.11)	0.389
- BMI +1 Unit	3.86 (1.29)	4.02 (1.15)	-0.12 (-0.34, 0.11)	0.313	-0.12 (-0.34, 0.11)	0.311
Thigh SFTM (mm)[b]				0.449*		0.439*
- BMI At Mean	6.69 (1.87)	7.09 (2.47)	-0.28 (-0.62, 0.06)	0.105	-0.29 (-0.63, 0.04)	0.087
- BMI +1 Unit	7.08 (2.16)	7.22 (2.27)	-0.20 (-0.60, 0.20)	0.319	-0.21 (-0.61, 0.18)	0.286
Birthweight <2.5kg[a]				0.341*		0.328*
- BMI At Mean	9 (5.59)	9 (5.75)	1.33 (0.69, 2.58)	0.397	1.34 (0.69, 2.60)	0.388
- BMI +1 Unit	11 (7.10)	6 (3.87)	1.60 (0.73, 3.51)	0.239	1.62 (0.74, 3.56)	0.227
LGA[a]				0.191*		0.200*
- BMI At Mean	12 (7.45)	9 (5.72)	0.89 (0.51, 1.55)	0.675	0.90 (0.51, 1.57)	0.703
- BMI +1 Unit	10 (6.45)	16 (10.32)	0.71 (0.38, 1.34)	0.294	0.72 (0.38, 1.36)	0.317
SGAa				0.533*		0.565*
- BMI At Mean	9 (5.59)	13 (8.28)	0.85 (0.48, 1.49)	0.569	0.86 (0.49, 1.51)	0.599
- BMI +1 Unit	12 (7.74)	12 (7.74)	0.94 (0.47, 1.87)	0.864	0.95 (0.48, 1.89)	0.878

[a] number and percentage of events for participants at or below the median BMI, and for participants above the median BMI. Estimates are Relative Risks and 95% Confidence Interval, for Lifestyle Advice vs Standard Care. Estimates of intervention effect were derived separately (a) for mean BMI, (b) corresponding to a 1 unit

increase in BMI, regardless of the signifi*cance of the interaction term.* [b] mean and standard deviation for participants at or below the median BMI, and for participants above the median BMI. Estimates are differences in means and 95% Confidence Interval (Lifestyle Advice – Standard Care). Estimates of intervention effect were derived separately (a) for mean BMI, (b) corresponding to a 1 unit increase in BMI, regardless of the significance of the interaction term. [c] Adjusted analyses: all outcomes were adjusted for the stratification variable parity (0 vs 1+), maternal age (continuous), and SEIFA IRSD Quintile. * Denotes p value for test of interaction between BMI and intervention, i.e. whether the effect of the intervention differs by maternal prepregnancy BMI. **=Includes all women randomized who did not withdraw consent to use their data, and who did not suffer miscarriage or termination of pregnancy prior to 20 weeks gestation, or stillbirth.
